# Efficacy and Toxicity of Immune -Checkpoint Inhibitors in Patients With Preexisting Autoimmune Disorders

**DOI:** 10.3389/fmed.2020.00137

**Published:** 2020-05-07

**Authors:** Michelle Coureau, Anne-Pascale Meert, Thierry Berghmans, Bogdan Grigoriu

**Affiliations:** ^1^Service des Soins Intensifs et Urgences Oncologiques, Institut Jules Bordet, Université Libre de Bruxelles (ULB), Brussels, Belgium; ^2^Service d'Oncologie Medicale, Unité d'Oncologie Thoracique, Institut Jules Bordet, Université Libre de Bruxelles (ULB), Brussels, Belgium

**Keywords:** cancer, immunotherapy, autoimmunity, side-effect, flare-up

## Abstract

Immunotherapy is an important armamentarium for cancer treatment nowadays. Apart from their significant effectiveness in controlling disease they also generate potential severe immune related adverse effects. Preexistence of immune related conditions may eventually predispose to the development of more severe complication and extreme caution have been taken in treating these patients. We performed a literature review searching for case reports and case series in order to offer evidence-based data for clinical management of these patients. Preexisting serological-only immune abnormalities or presence of a predisposing genetic background does not seem to confer significant risk but existing data is scarce. Most patients with preexistent autoimmune diseases can probably treated with checkpoint inhibitors as they seem to have at least the same response rate as the general cancer population. Under treatment, a significant part of them (at least 30%) can experience a flare of their baseline disease which can sometime be severe. Life-threatening cases seems rare and disease flare can be generally managed with steroids. The volume of available data is more important for rheumatologic diseases than for inflammatory bowel diseases were more caution should be observed. However, it has to be kept in mind that new immune related adverse effects (IrAE) are seen with a similar frequency as the flare of the baseline disease. Both flare-up's and newly developed IrAE are generally manageable with a careful clinical follow-up and prompt therapy.

## Introduction

Immunotherapy has become a cornerstone in cancer treatment offering unexpected treatment opportunities for patients with metastatic, loco-regional, or even operable tumors in melanoma, lung, urothelial, and a number of other tumoral localizations.

Two main directions of intervention are now clinically available for manipulating immune checkpoints by either targeting CTLA-4 or PD-1/PD-L1. Our current understanding of the role of these two molecules is that anti CTLA-4 is involved in the lymphocyte proliferation process after antigen specific T-cell receptor activation while anti PD-1/PD-L1 act predominantly in peripheral tissues during the effector step. However, CTLA-4 is also expressed on regulatory T lymphocytes and is thus involved in peripheral inhibition of T cell proliferation.

Despite the effectiveness of these products, two main issues arise from theoretical and practical considerations.

First not all patients respond to therapy. To date prediction of response to immune checkpoint inhibitors is difficult and available predictive markers are not optimal. Secondly cancer may be associated with a number of autoimmune manifestations either as a paraneoplastic condition (1) or as comorbidities. In experimental models, knocking-out CTLA-4 results in a massive proliferation and lymphocytic infiltration of tissues (2) while PD-1 knock-down mice are prone to autoimmune disease [reviewed in (3)]. These phenotypes can also be found in humans with CTLA-4 (4, 5) or PD-L1 (6) deficiency. Nevertheless, the mechanisms involved in antitumor immune response induced by checkpoint inhibitors may be different from those of autoimmune disease. However, it is clear that using checkpoint inhibitors to treat cancer poses the risk of a flare up of preexistent autoimmune disease and the development of new autoimmune manifestations in patients with preexisting autoimmunity even if present as a biological only manifestation (as for example positive auto antibodies).

Thus, these patients with preexisting autoimmune manifestations have been traditionally excluded from clinical trials as it was expected to have severe autoimmune manifestations which could exceed the potential benefit from tumor control. This subgroup of cancer patients harboring autoimmune manifestations is not trivial. Depending on the definition used, a large register-based analysis (SEER) including 2,10,509 patients with lung cancer identified between 13.5% (more restrictive definition) and 24.6% (more liberal definition) of cancer patients as having an autoimmune disease of any type (7). The most frequent manifestations found were rheumatoid arthritis (5.9%), psoriasis (2.8%), polymyalgia rheumatica (1.8%), Addison disease (1%), systemic lupus erythematosus (SLE-−0.9%), ulcerative colitis (0.8%), giant cell arteritis (0.8%), sicca syndrome (0.6%), regional enteritis (0.5%), and Meniere disease (0.5%). However, these autoimmune manifestations can be very broad as it has been recently reviewed for lung cancer in a series of articles curated by Jean Paul Scuiler (1, 8–11).

Thus, it is useful to perform, prior to any treatment, a careful evaluation of the possible autoimmune comorbidities in order to avoid possible harm, evaluate risk/benefit ratios, and anticipate possible complications.

## Patients at Risk but Without Previous Clinical Evidence of Autoimmune Disease

In patient with lung cancer receiving checkpoint inhibitors, development of diverse IrAE is not completely random. Despite the fact that the incidence of IrAE increase with treatment duration, there is a pattern of developing specific organ manifestations in defined time frames. Thus, one can postulate that these IrAE develop in patients caring specific genotypes or having a previous immune priming predisposing for developing IrAE. Very little is known on this matter. Hasan Ali et al. (12) reported on a small series of 102 patients receiving anti PD-1/PD-L1 and anti CTLA-4 therapies, a very strong correlation between the presence of pruritus and the HLADRB1^*^11:01 allele. Patient carrying these allele not only develop more pruritus than controls but also developed it earlier. Interesting, in previous investigations this specific allele has been associated with the development of atopic dermatitis in a small cohort of 185 children of Korean origin (13). The same allele has been associated with systemic sclerosis with anti-topoisomerase antibodies (14) or with the risk of developing systemic juvenile arthritis (15) or atopic dermatitis (13). The same Swiss group of Hasan Ali et al. also demonstrated an association trend between HLA DBQ1^*^03:01 allele and the development of colitis. Again, this allele has been previously associated firmly with the development of inflammatory bowel disease (16). Similarly it is possible that some Single Nucleotide Polymorphism (SNP's), which are associated with a higher risk of autoimmune disease (colitis, diabetes, and allergy), positively impact the response rate to anti PD-1 therapy (17).

A systematic review of susceptible loci associated with development of IrAE's under checkpoint inhibitor therapy has been recently performed by Hoefsmit et al. (18) with a compartmentalized analysis based on affected organ and type of adverse effect. A huge number of susceptible loci, with a very variable physiologic/pathologic role, have been identified. However, these data can be hypothesis generating at most. At present time there is no sound information that can be applied to practice and there is no scientific basis to withhold checkpoint inhibitor treatment in people considered at risk based on these criteria.

Yoneshima et al. analyzed if pretreatment positive antinuclear antibodies (ANA) are associated either with IrAE or treatment response after administration of PD-1 inhibitors (19). They analyzed a group of 83 patients, of which 18 had preexisting positive serum ANA at a titer of 1:40 or higher. There was no statistical relationship between the presence of ANA and the risk of developing IrAE but there was an association trend between increasing ANA titers and the development of IrAE. Overall, response and disease control rates were similar in patients with or without ANA but there was also a higher PFS and OS in patients with negative ANA. A similar investigation was conducted by Sakakida et al. (20) which followed 191 patients treated with nivolumab, pembrolizumab, atezolizumab, or durvalumab and used a cutoff titer for positive ANA at 1:160. There was no significant difference in the development of IrAE between ANA positive and ANA negative patients with the exception of patients who developed colitis which had higher ANA titers than those who do not. Again, an elevated ANA titer was associated with a numerically lower disease control rate (37.5 and 67.5%) but this was not statistically significant (*p* = 0.08). Furthermore, for both series, the global numbers are rather small and the significance (both from statistical and clinical point of view) is debatable. Moreover, there are case reports of serious IrAE (i.e., fatal pneumonitis) that followed immune checkpoint administration in patients with pre-existing anti-synthetase syndrome (21).

To date, there is no sufficient data to warrant a systematic search for autoantibodies in cancer patients that are planned to receive checkpoint inhibitors. However, if knowledge of such positive finding is available, a clinical surveillance is warranted in order to identify, as early as possible, autoimmune manifestations associated with these autoantibodies.

## People With Preexisting Clinically Diagnosed Autoimmune Disease

Since these patients have been systematically excluded from clinical trials, subgroup analysis is scarcely available in the literature. Existing data originate from case reports, retrospective analyses of small series or registry data. Only one subgroup analysis of randomized trials is available but it does not cover the entire spectrum of autoimmune disease encountered in practice.

### Case Reports

A number of 37 publications reporting 41 cases have been found [(22–59); Table 1]. Most cases were metastatic melanomas (*n* = 32), five non-small cell lung cancers, two Merkel cell carcinoma, one urothelial carcinoma, one colon cancer. These 41 cases received 44 immunotherapy treatments namely ipilimumab (*n* = 15), one reinduction ipilimumab, nivolumab (*n* = 9), or pembrolizumab (*n* = 18), one unclear, which globally reflect the successive market authorizations of these drugs. No reports combining immunotherapy with chemotherapy were found. Pre-existing autoimmune manifestations consisted of Crohn disease/ulcerative colitis (*n* = 8), rheumatoid arthritis (*n* = 7), psoriasis (*n* = 8), myasthenia gravis (*n* = −6), multiple sclerosis (*n* = 3), Hashimoto thyroiditis (*n* = 2), and one each of lupus, sarcoidosis, immune thrombocytopenia, melanoma associated retinopathy, Churg Strauss syndrome, granulomatosis with polyangiitis, hypothyroidy and type 1 diabetes, Bechet disease, and bullous pemphigoid. Globally a tumor effect of immunotherapy is reported for 40 out of the 44 treatments with six complete responses, 27 partial responses, three stable disease and four progressions. The response rate is 82.5% and the disease control rate is 90% which are very high figures but is it highly probable that this reflects a strong publication bias. In a majority of cases (*n* = 29, i.e., 65.6%) immunotherapy resulted in a flare up of the baseline disease which was managed with steroids or infliximab, adalimumab, or rituximab. In 10 cases (i.e., 22.7%) flare ups were considered severe/very severe with one death. In another case, a flare up of colitis was infliximab resistant and the patient subsequently developed a toxic epidermal necrosis and died. Only 16 cases had no flare up of the base line disease. Six of these patients developed other IrAE as pneumonitis, toxic epidermal necrosis, acute colitis, psoriasis, vasculitic neuropathy or acute interstitial nephritis. Overall, mortality due to disease flareup is about 4.5% and very severe (grade 4) toxicities unresponsive to steroids or even additional anti TNF alpha therapy was not uncommon. In only 12 cases no flare-up and no subsequent IrAE developed.

**Table 1:** List of case report of Checkpoint inhibitors in patients with preexisting autoimmune diseases.

**References**	**Pre-existing autoimmune disease**	**Immunosuppressive treatment**	**Cancer type**	**Drug received**	**Response**	**Disease flare Yes/No new autoimmune manifestation (treatment)**
Gerdes et al. (31)	Multiple sclerosis	No (minimal radiologic only disease)	Melanoma	Ipi	Yes (PR)	Yes (massive flare)
Gettings et al. (32)	Multiple sclerosis	–	Melanoma	Ipi	Yes (PR)	Yes (Severe Flare)
Kyi et al. (38) *N* =2	Multiple sclerosis	IFN beta	Melanoma	Ipi	No	No
	Rheumatoid arthritis	MTX +PDN	Melanoma	Ipi	Yes (PR)	No
Benson et al. (25)	Rheumatoid arthritis	Etanercept	Melanoma	Ipi	No	Yes Acute interstitialnephritis
				Pem	Yes (PR)	Yes (hydroxychloroquine +prednisone)
Puri and Homsi (48)	Rheumatoid Arthritis	PDN low dose	Melanoma	Pem	Yes (CR)	No
Aya et al. (23)	Rheumatoid arthritis	No	Melanoma	Ipi	Yes (CR)	No Colitis(infliximab)
				Pem	Yes (PR)	No Vasculiticneuropathy
Hedge et al. (34)	Rheumatoid arthritis	No	Melanoma	Pem	Yes (PR)	–
Kageyama et al. (35)	Rheumatoid arthritis	Salazopyrine	Melanoma	Nivo	Yes (CR)	No
Zhu and Li (57)	Rheumatoid arthritis and Myasthenia gravis	No	Melanoma	Pem	Yes (PR)	Yes (IvIg, plasmapheresis, PDN)
Zaremba et al. (56)	Myasthenia gravis	AZT (followed by MMF and cyclosporine)	Merkel cell carcinoma	Pem	Yes (PR)	No
Lau et al. (39)	Myasthenia gravis	AZT	Melanoma	Pem	–	Yes (steroids + IvIg)
Maeda et al. (40)	Myasthenia gravis	PDN	Melanoma	Nivo	Yes (PR)	Yes (Self-limited)
Cooper et al. (28)	Myasthenia gravis	No (pyridostigmine)	NSCLC	Nivo	Yes (PR)	Yes (severe – death)
Phadke et al. (46) *N* =2	Myasthenia gravis	MMF	Melanoma	Pem	Yes (PR)	Yes (Grade 4)
	Psoriasis/psoriatic arthritis	MTX	Melanoma	Pem	Yes (CR)	Yes
Roche et al. (49)	Psoriasis/psoriatic arthritis	–	Merkel cell carcinoma	Pem	Yes (PR)	No
Sahuquillo-Torralba et al. (50)	Psoriasis	Local treatment Psoriasis severity score 3 on 5% onBSA	NSCLC	Pem	Yes (PR)	Yes (severe – Psoriasis score 22 on 81% of BSA; Pem continued and resolution with severity score 4 after acitretin 35 mg/d)
De Bock et al. (58)	Psoriasis	No	Melanoma	Nivo (previous Ipi)	Yes (SD)	Yes (local steroids and every 3 week Nivo)
Chia and John (27)	Psoriasis	No	NSCLC	–	–	Yes (severe flare)
Kato et al. (37)	Psoriasis	No	Melanoma	Nivo	–	Yes
Matsumura et al. (41)	Psoriasis	No	Melanoma	Nivo	Yes (PR)	Yes Pneumonitis
Esfahani and Miller (29)	Psoriasis+ Crohn disease	No	Colon	Pem	Yes (PR)	Yes Psoriasis and gastrointestinal
Frohne et al. (30)	Crohn disease	Infliximab/AZT followed by Vedolizumab	Melanoma	Pem	Yes (CR)	No
Uemura et al. (53)	Crohn disease	Tocilizumab	Melanoma	Pem	Yes (PR)	Yes (adalimumab)
Gielisse and de Boer (33)	Crohn disease	No	Melanoma	Ipi	Yes (PR)	Yes (manageable)
Kamil et al. (36)	Crohn disease	Mesalazine and PDN	NSCLC	Ipi	Yes (PR)	Yes (infliximab resistant) Toxic epidermal necrosis (death)
Bostwick et al. (26)	Ulcerative colitis	No	Melanoma	Ipi	Yes (PR)	Yes (infliximab)
		AZT+ PDN		Reinduction Ipi	Yes (CR)	Yes Also new auto immunedisease
Pedersen et al. (45) *N* =2	Ulcerative colitis	Infliximab	Melanoma	Ipi	Yes (PR)	No
	Bechet disease	–	Melanoma	Ipi	No	No
Plachouri et al. (47) Wieshaupt and Sunderkötter (54) (duplicatepublication)	Sarcoidosis	PDN low dose	Melanoma	Ipi	No	Yes (Flare with muscular involvement)
Audemard et al. (22)	Melanoma Associated Retinopathy	No	Melanoma	Ipi	Yes (PR)	No (diminishing symptoms secondary to response)
Beck et al. (24)	Bullous pemphigoid	No	Melanoma	Ipi	Yes (SD)	Yes – steroids
				Pem	Yes (SD)	Yes – steroids
Maul et al. (42)	Churg Strauss + Ipi induced colitis	–	Melanoma	Pem	Yes (PR)	No
Nabel et al. (43)	GPA (granulomatosis with polyangiitis)	Cyclophosphamide PTX, PDN	MEN2+ Urothelial carcinoma	Pem	Yes (PR)	Yes: PDN +rituximab
Tagliamento et al. (52)	SLE + HCV	PDN 5 mg HCQ 200mg	Melanoma	Nivo	Yes (PR)	No
Stephen Bagley et al. (51)	Immune thrombocytopenia	No	NSCLC	Nivo	Yes (PR)	Yes (mild)
Akturk et al. (59)	Hypothyroidism Type 1 diabetes (+renal graft)	Immuno suppression (PDN, MMF, Tacro—graft)	Melanoma	Pem + Nivo	–	Yes (graft rejection) Diabetic ketoacidosis and more severe hypothyroidia
Narita et al. (44) *N* = 2	Hashimoto thyroiditis	No	Melanoma	Nivo	Yes (PR)	Yes (serological)
Yonezaki et al. (55)	Hashimoto thyroiditis	–	Melanoma	Ipi (after Nivo)	Yes (PR)	Yes thyroid storm

*HCQ, hidroxycloroquine; PDN, prednisone; AZT, azathioprine; MMF, mycophenolate mofetil; CycloA, ciclosporin A; Nivo, nivolumab; Pem, pembrolizumab; Ipi, ipilimumab; HCV, hepatitis virus C; SLE, systemic lupus erythematosus*.

### Data From Clinical Trials

Even if patients with previous autoimmune disease have been excluded from clinical trials a number of patients have been nevertheless included. The FDA made a *post-hoc* analysis based on aggregate data from 22 trials with four Anti PD-1/PDL-1 agents that was published only in an abstract form (60). The series mainly include thyroid disease and cutaneous autoimmune manifestations (psoriasis and vitiligo)-−54.5% of cases. Worsening of the underlying disease occurred in 6–16 percent of the cases. Two grade 4 hyperglycemia were found in patients with preexisting diabetes, as well as three grade 3 hypothyroidy, psoriasis, and interstitial lung disease and one grade 3 ankylosing spondylitis. Only 8–9% of cases needed steroid prescription and duration of treatment varied between 145 and 196 days. Thus, in this population with probably a strong bias toward non-severe autoimmune manifestations, there are no consequent safety signals identified and administration of PD-1/PDL-1 inhibitors seems safe.

However, the report lacks data concerning the exact type of autoimmune condition for a large proportion of patients. Data on severity and treatment of baseline disease is also scarce and thus these results are to be interpreted with caution.

### Case Series

We found five retrospective series in patients with melanoma (61–65) totalizing 150 cases with autoimmune disease previous to any treatment and 117 with autoimmune manifestations due to a previous therapy with ipilimumab which received another checkpoint inhibitor afterwards. In the series receiving only ipilimumab (61, 63, 64) the response rates varied between 12 and 50% (higher for smaller series) while in series with anti PD-1/PDL-1 (62, 65) the response rate was around 30% which is in line with what was described with these drugs in clinical trials. Between 27 and 50% of cases had a flare up of their baseline autoimmune condition while up to 30% of patients experienced the development of a new autoimmune toxicity. Grossly, the frequency of flare ups is similar to that of newly developed immune related manifestations. Severity of flare-ups was generally moderate, with 50% being more than grade one and no very severe cases (grade 4/5). Interestingly, in cases with an ipilimumab induced autoimmune disease, a flare up is rarely seen when subsequently treated with a PD-1/PDL-1 inhibitor. It seems that the presence of an autoimmune manifestation under CTLA-4 inhibition does not preclude anti PD-1/PDL-1 treatment but data in this area is still scare in order to make a firm clinical recommendation. Two case series of the same author (66, 67) reported 100 NSCLC cases and another one (68) 112 cases in multiple tumor types.

In these three series the tumor response rates varied between 45 and 51% while disease flare is seen in 17–47% of patients. Most of flare-ups are moderate (grade 3 or less). Between 25 and 42% of patients experienced a new IrAE, not related to the baseline autoimmune disease, during the course of treatment.

The reports do not always include detailed data concerning treatment received for the baseline autoimmune disease and thus it is difficult to derive clinical rules on which patients would eventually develop or not a flare-up of the baseline disease.

A number of 5 cases series with matched controls receiving the same immune checkpoint inhibitor but without autoimmune disease were published (69–73); these series included a total of 701 patients with a very large panel of autoimmune disorders (Table 2). Concerning antitumor efficacy, there was no difference between patients with or without previous autoimmune disease (70, 73). Up to 47% of patients experienced a flare up of their baseline disease and up to 42% developed a new IrAE. These figures, of newly generated IrAE, are comparable with those reported from randomized clinical trials (74). Severe and very severe cases (grade 3 and up) were not reported more frequently.

**Table 2:** Case series with preexisting autoimmune disease (AD) treated with checkpoint inhibitors.

**References**	**Tumor**	***N* =**	**Baseline autoimmune dis**	**Drug**	**Benefit**	**Toxicity**
***Post-hoc*** **analysis of clinical trial data**						
Weinstock et al. (60)	All tumors	*N* = 552	Thyroid *n* = 188–34% Psoriasis *n* = 70–12.5% Vitiligo *n* = 44–8% Other = 250	Anti PD-1/PDL-1 (no details)	No data (mean duration of treatment between 145 and 196 days)	Worsening of baseline disease between 6 and 16%. Two grade 4 hyperglycemia and 7 grade 3. Steroids required in 8–9% of patients 17% experienced anyIrAE
**Retrospective case series**						
Lee et al. (61)	Melanoma	*N* = 8	RA (*n* = 8) 50% MTX and/or AntiTNFa	Ipi	2 CR (25%) 2 PR 3 SD 1 PD 4 responses at 11m	62.5% treat discontinuation Median 2 cycles Dis flare 50% grade 1 25% 50% more than grade 1 Grade 3 IrAE (All G3colitis)
Johnson et al. (63)	Melanoma	*N* = 30	Rheumatoid arthritis *n* = 6 Crohn/ulcerative colitis *n* = 6 Psoriasis *n* = 5 Multiple sclerosis *n* = 2 Lupus *n* = 2 Sarcoidosis *n* = 2 Thyroiditis *n* = 3 Other *n* =7	Ipi	PR + CR *n* = 6 (20%) Stable disease *n* = 3(10%)	27% had a flare -up No severe cases 33% had an IrAE (Colitis, Thyroiditis, Hypophysitis)
Kahler et al. (64)	Melanoma	*N* = 41 (with 44 AD conditions)	Thyroiditis *n* = 15, rheumatoid *n* = 11, dermatologic *n* = 10, Crohn's disease/ulcerative colitis *n* = 3, neurological *n* = 2, sarcoidosis *n* = 2, pancreatitis *n* = 1 *n* = 11 on immunosuppressors(25%)	Ipi	1 CR, 4 PR ≥ 12% response rate	*N* = 12 (29.2%) resulted in a flare up of preexisting disease *N* = 12 (29.2%) had a new IrAE *N* = 23 (56%) had no flare/no new IrAE
Menzies et al. (62)	Melanoma	*N* = 119 95 with IrAE after Ipi *N* = 52 preexisting AD	Not reported 29% AD active symptoms	Pem 91.5% Nivo 8.5%	Response rate 33% in preexisting AD and 40% if IrAE on Ipi	Flare up in 38%: -RA 54% -Polymyalgia 100% -SJögren, thrombocytopenic purpura, 100% -No flare if Crohn or neurological disease 29% developed new adverse effects No deaths
Gutzmer et al. (65)	Melanoma	*N* = 19	Thyroiditis *n* = 6 Psoriasis *n* = 3 Spondylarthritis/ankylosing spondylitis/ Myositis/polymyalgia/rheumatoid arthritis *n* = 6 Sarcoidosis *n* = 2 Multiple sclerosis/Guillain Barré/Ulcerative colitis/Churg Strauss *n* = 1each	Nivo 63% Pem 37%	32% PR 10% SD 58% PD	42% had flare up in 3–20 weeks -Rhumatological disease: 55% -Colitis 100% -Psoriasis 50% -Thyroiditis20% 16% had a new IrAE
		*N* = 22	IrAE induced by Ipi Colitis *n* = 11 (Gr 3 = 7 GR4 = 1) Hypophysitis *n* = 10 (Gr3 = 4) Thyroiditis *n* = 3 Hepatitis *n* =1	Nivo 41% Pem 59%		Only 1/22 Flare-up (5%) 23% developed another IrAE
Leonardi et al. (66)	NSCLC	*N* = 46	Not reported 13% active disease 19% receive immune treat	Anti PD-1 (no details)	No data	17% flare of baseline disease 26% developed other IrAE No grade 4–5
Leonardi et al. (67)	NSCLC	*N* = 56	Rheumatologic *n* = 25(45%) Dermatologic *n* = 16 (29%) Endocrine *n* = 9 (16%) Crohn/ulcerative colitis *n* = 6 (11%) Myastenia/Multiple sclerosis *n* = 3(5%)	Nivo 80% Pem 18% Atezo 2%	PR 22% SD 31% PD 47% No relation of flare or steroids at beginning of treatment with response	23% flare of baseline disease -13% grade 3, 0% Grade 4 and 5 -50% of symptomatic had a flare vs. 18% of asymptomatic patients -Rheumatologic patients 40% flare vs. 10% other 38% developed another IrAE 5% developed both
Tison et al. (68)	Melanoma59% NSCLC 35% Urothelial 3% MCC 1.5%	*N* = 112	Psoriasis *n* = 31 (28%) Rheumatoid arthritis *n* = 20 (18%) Inflammatory bowel dis *n* = 14 (13%) Spondilartritis *n* = 5 (4,5%) Lupus *n* = 7 (6,3%) Polymyalgia/Gian cell arteritis *n* = 7 Other *n* = 28(25%)	Ipi *n* = 13% Nivo 56% Pem 24% Ipi + Nivo 3% Atezo 2% Ave 2%	30% PR 15%CR	47% flare of baseline disease -70% mild, 30% severe -45% required steroid -9%other immunosuppression 42% another IrAE -60% mild 40% severe 18% had both flare + new IrAE 1 death
**Series including a control group with ICI treated patients**						
Danlos et al. (69)	Melanoma 80% NSCLC 13.3% Other 6.7%	*N* = 45 vs. 352 controls	Rheumatic disease, *n* = 7 (13%) Dermatologic disease, *n* = 33 (62%) Endocrine disease, *n* = 9 (17%) Neurologic disease, *n* = 3 (5.7%) Hematologic disease, *n* = 1 (1.9%) Symptomatic stable disease 50.9% Flare (at ICI initiation) = 5.7% 6 ICI pretreated experienced colitis (*n* = 3), thyroiditis, hypophisitis, and nephritis	Pem 75.6% Nivo 22.2% Ave 2.2% 9 patients pretreated with AntiCTLA-4/PD-1	Not reported	20 (44.4%) experienced and IrAE Of which 11 (55%) where flare UP (i.e., *n* = 11/45–24.5%) 10 patients developed a new IrAE Only in 5 of the 20 patients ICI treatment was stopped After excluding vitiligo 51,6 patients did not develop an IrAE under ICItreatment
Cortellini et al. (70)	NSCLC 65.5% Melanoma 21% RCC 12.5% Other1%	*N* = 85 vs. 666 controls	Thyroid disorder *n* = 51 (60%) Dermatologic *n* = 14 (16.4%) Rheumatologic *n* = 10 (11.8%) Gastrointestinal/hepatic *n* = 4 (4.7%) Neuro-/Nephro- *n* = 1 (1.2%) each Multiple *n* = 4(4.7%)	No data	Response rate 35,3% No difference with or without AD	With preexisting AD: -All grade 65.9% (95%CI 49.7–85.5) -G3/G4 9.4% (95%CI 4.1–18.5%) -Flare of baseline Disease 47.1% -No Diff between active and inactive disease at baseline Without preexisting AD -All grades 39,9% (95%CI 35.2–45) -G3/G4 8.8% (95%CI6.7–11.4)
Ricther et al. (71)	Melanoma 63% NSCLC 25% Hematologic13%	*N* = 16 (out of 33 with preexisting AD and 700 ICI treated patients)	Polymyalgia rheumatica *n* = 5 (31%) Rheumatoid arthritis *n* = 5 (31%) Giant Cell arteritis *n* = 2 (13%) Sjogren syndrome *n* = 2 (13%) Lupus *n* = 2 (13%) Ankylosing spondylitis *n* = 1 Idiopathic aortitis *n* = 1 Sarcoidosis *n* = 1 Urticarial vasculitis *n* =1	Ipi 31% Nivo 44% Pem 31%		IrAE developed in *n* = 6 (38%) Colitis *n* = 3 Only 1 case had a flare of baseline Rheumatic Disease 5 of 6 occurred during first 4 cycles 4 of 6 were grade 3 or 4 All managed with steroids. One treated with another IC with no relapse
Khozin et al. (73)	NSCLC	538 with AD out of 2,425 patients	Not reported	Not reported	No difference for PFS, OS, Time to treatment discontinuation and time to next treatment	No increasing IrAE in patients with preexisting autoimmune disease
Shah et al. (72)	Melanoma 45% Lung Cancer 18% Other 37%	*n* = 22 with AD out of 638	Hypothyroidism (36.4%) Graves disease (9%) Psoriasis, vitiligo (*n* = 2–9%) each Ulcerative colitis (*n* = 2–9%) Type 1 diabetes, ankylosing spondylitis, lupus, nephropathy (*n* = 1–4.5%each)	Ipi 45% Pem 41% Nivo 27%	No data	Only *n* = 4 (18%) had an exacerbation of previous Autoimmune disease 19 out of 22 had a new IrAE Pulmonary *n* = 10 Gastrointestinal *n* = 6 Heptic *n* = 5 Renal *n* = 3 Hypothyroidism *n* = 2 Dermatological *n* = 2 Musculoskeletal *n* = 2 Hypophisitis *n* =1
Kehl et al. (75)	Lung cancer 42% Melanoma 34% Renal 9% Urothelial 3.5% Head and neck 5% Other6.5%	179 (strict criteria) Out of 4,438 treated patients	Not reported	Nivo 52% Pem 20% Ipi 21% Ipi + Nivo 5% Atezo 3%	Mean treatment duration 13.7 weeks	Pre-existing autoimmune disease not related with all cause hospitalization but related with hospitalization for an IrAE (HR 1.81 95%CI 1.21–2.71) and prescription of steroids (HR 1.93 95%CI 1.35–2.76)

Finally, an analysis of the SEER registry data (75) showed that a preexisting autoimmune disorder is not related to all cause hospitalization but is associated with hospitalization related to the development of IrAE and with steroid prescription.

## Conclusions

While it is difficult to aggregate such very disparate data some conclusion may still be extracted. First, patients with autoimmune disorders seems to derive the same benefit in term of tumor response compared with people without autoimmune disorders. Conflicting data exist concerning the association between autoimmunity or IrAE and tumor response, with both positive and negative investigations (76–79). Much more research is needed in this area. Thus, there is no an “a priori” reason to deny a potential effective treatment to these patients. Weather this group of patients experience more long-term benefit is still a matter of investigation.

Concerning safety, these patients seem to generate the same spectrum, number, and severity of IrAE (other than the flare up of their baseline autoimmune condition) as patients without preexisting autoimmune disease and thus need to be followed-up similarly. The question arises for the flare-up of their autoimmune condition. At least 30% to a maximum of 80% of patients, depending on series and type of baseline autoimmune disease, do not experience a flare up. In general, people with skin autoimmune disorders can be managed safely even in case of very severe flares (50). The same apply for those with thyroid dysfunction but severe hyperthyroidism can be encountered even in previously chronic hypothyroid patients (55).

Some data exist for patients with rheumatic disease, and it seems that at least 50% of them experience a disease flare. Grade 4 and 5 flare-ups seems infrequent and generally are manageable with steroids and anti-TNF medications. These patients can receive checkpoint inhibitors provided that no best option is available, they are informed on the potential disease flare and a careful follow-up is instituted.

Insufficient data exist for patients with inflammatory bowel disease. When these manifestations develop during a previous anti CTLA-4 treatment, the relapse rate under anti PD-1/PDL-1 therapy seems low and it is probable that these patients can be safely treated.

There is a need for more research in groups of patients with inflammatory bowel disease which receive checkpoint inhibitors for the first time. Their administration clearly poses some risks, but it needs to be evaluated taking into account the effectiveness of available alternatives and the patient should understand the potential benefits and harms as well as the risk -benefit ratio.

Based in SEER data, administration of immune checkpoint inhibitors do not result in excess hospitalizations compared to similar patients without autoimmune disease but the hospitalizations are strongly related to the development or flare up of autoimmune conditions. An observational trial concerning ipilimumab in patients with melanoma and underlying autoimmune diseases (80) has been performed but, to our best knowledge, results have not yet been reported.

## Author Contributions

All authors contributed to literature search, data extraction, and drafting of manuscript.

## Conflict of Interest

The authors declare that the research was conducted in the absence of any commercial or financial relationships that could be construed as a potential conflict of interest.
